# Study Protocol: Interactive Dynamics of Coral Reef Fisheries and the Nutrition Transition in Kiribati

**DOI:** 10.3389/fpubh.2022.890381

**Published:** 2022-06-01

**Authors:** Christopher D. Golden, Julien Ayroles, Jacob G. Eurich, Jessica A. Gephart, Katherine L. Seto, Michael K. Sharp, Prentiss Balcom, Haley M. Barravecchia, Keegan K. Bell, Kelvin D. Gorospe, Joy Kim, William H. Koh, Jessica Zamborain-Mason, Douglas J. McCauley, Helen Murdoch, Nilendra Nair, Kaaro Neeti, Simone Passarelli, Aaron Specht, Elsie M. Sunderland, Aritita Tekaieti, Aranteiti Tekiau, Rosemary Tekoaua, Eretii Timeon

**Affiliations:** ^1^Department of Nutrition, Harvard T.H. Chan School of Public Health, Boston, MA, United States; ^2^Department of Environmental Health, Harvard T.H. Chan School of Public Health, Boston, MA, United States; ^3^Department of Ecology & Evolutionary Biology, Princeton University, Princeton, NJ, United States; ^4^Marine Sciences Institute, University of California, Santa Barbara, Santa Barbara, CA, United States; ^5^Environmental Defense Fund, Santa Barbara, CA, United States; ^6^Department of Environmental Science, American University, Washington, DC, United States; ^7^Department of Environmental Studies, University of California, Santa Cruz, Santa Cruz, CA, United States; ^8^Statistics for Development Division, Pacific Community, Noumea, New Caledonia; ^9^Australian National Centre for Ocean Resources and Security, University of Wollongong, Wollongong, NSW, Australia; ^10^School of Engineering and Applied Sciences, Harvard University, Cambridge, MA, United States; ^11^BAO Systems, Washington, DC, United States; ^12^Ministry of Health and Medical Services, Tarawa, Kiribati; ^13^National Statistics Office, Ministry of Finance and Economic Development, Tarawa, Kiribati; ^14^Ministry of Fisheries and Marine Resources Development, Tarawa, Kiribati

**Keywords:** food security, planetary health, small island developing state (SIDS), diabetes, obesity, hypertension, social-ecological traps, traditional diets

## Abstract

The Kiribati 2019 Integrated Household Income and Expenditure Survey (Integrated HIES) embeds novel ecological and human health research into an ongoing social and economic survey infrastructure implemented by the Pacific Community in partnership with national governments. This study seeks to describe the health status of a large, nationally representative sample of a geographically and socially diverse I-Kiribati population through multiple clinical measurements and detailed socio-economic surveys, while also conducting supporting food systems research on ecological, social, and institutional drivers of change. The specific hypotheses within this research relate to access to seafood and the potential nutritional and health benefits of these foods. We conducted this research in 21 of the 23 inhabited islands of Kiribati, excluding the two inhabited islands—Kanton Islands in the Phoenix Islands group with a population of 41 persons (2020 census) and Banaba Island in the Gilbert Islands group with a population of 333 persons (2020 census)—and focusing exclusively on the remaining islands in the Gilbert and Line Islands groups. Within this sample, we focused our intensive human health and ecological research in 10 of the 21 selected islands to examine the relationship between ecological conditions, resource governance, food system dynamics, and dietary patterns. Ultimately, this research has created a baseline for future Integrated HIES assessments to simultaneously monitor change in ecological, social, economic, and human health conditions and how they co-vary over time.

## Introduction

Globally, aquatic food systems support livelihoods and nutrition for billions of people (1). These benefits are derived both directly (e.g., consuming aquatic foods for better nutrition or catching and selling aquatic foods for income) and indirectly (e.g., using income derived from fishing livelihoods to support better nutrition). Yet, questions remain about the sustainability of aquatic food systems and their ability to continue delivering benefits to people (2). Approximately 1 billion people rely substantially on aquatic foods and could be placed at increased risk of nutritional deficiencies with ongoing environmental and socio-economic change (3). Quantifying the nutritional impact of different sources of environmental change (e.g., unsustainable fishing, climate change, coral bleaching), however, has been difficult in part because these system dynamics are complex, containing multiple direct, and indirect pathways and feedbacks.

We applied a social-ecological systems (SES) framework to study how feedbacks and interactive dynamics across social and ecological dimensions of coral reef food systems lead to differences in nutritional ecology [sensu (4)] of malnutrition and diet-related disease (5). In this system, we believe that reinforcing social and environmental changes have led to changes in food systems, and in turn, have led to changes in human health outcomes. We focused on reef-based food systems in Kiribati as a case study for several reasons. Seafood consumption in Kiribati is ~63 kg/person/year (6)—one of the highest in the world, and with high dependence on reef-based resources (7). In systems where aquatic foods are critical for nutrition and aquaculture is absent (8), such as nutritionally vulnerable countries, small-scale fisheries are a key sector to meet many dietary requirements. Reef-based foods are part of small-scale fisheries, and thus almost entirely retained in domestic markets (9, 10).

The geographic isolation of the country (and even greater isolation of some of its constituent islands) allowed us to examine the role of globalization and market integration in shaping food system dynamics in a more controlled way. Due to Kiribati's geographic diversity, a range of market, governance, and ecological circumstances exist, allowing for strong inter- and intra-island comparisons within the same national context.

### Environmental Change in Kiribati

Due to the geological constraints of low-lying coral reef atolls, Kiribati relies heavily on its marine resources for food security. With a lack of arable land, people depend on local reef and pelagic fisheries as a source of nutrition. However, anthropogenic influences have placed significant pressure on the nation's immediate coastal marine environment (11). The nation ranges from densely populated, urban islands with heavy reef degradation to sparsely populated and remote outer islands with intact marine systems e.g., 5,200 people/km^2^ in South Tarawa as compared to 300 people/km^2^ in South Tabiteuea (12, 13). This gradient of human pressure provides a lens to assess disturbances to reef systems. Local drivers of environmental degradation, such as fishing pressure, reef rock mining, land-use changes (e.g., sedimentation due to shoreline manipulation), nutrient run-off (e.g., sewage), and pollution, showcase the effects of population growth in urban centers (12). These patterns will likely increase with efforts to supply seafood to the burgeoning demand in domestic urban markets (14), as even urban islands like South Tarawa produce nearly half of their seafood from reefs. Specifically, signs of overexploitation in the lagoon and fore reefs from artisanal and subsistence fisheries can be seen around the main communities of South Tarawa (15), with surrounding islands beginning to lose flagship ecological indicators of a healthy reef system (see Data Analysis for a comprehensive description).

### Climate Change in Kiribati

As with many other low-lying atoll nations, climate change is forecasted to create a multitude of development and adaptation challenges in Kiribati. The magnitude and timing of these local impacts and the best adaptation pathways to address these global climate stressors are complex (16–18). Specifically, with increasing sea levels (14) and increasing frequency and severity of strong westerlies during El Niño Southern Oscillation (ENSO) episodes (19), storm surges have been more common (12) resulting in seawater contamination of freshwater resources (20) and sewage and fertilizer runoff into coastal areas. Further, Kiribati climate models indicate with “very high confidence” that both sea surface and air temperatures are increasing and will continue to do so throughout the twenty-first century (21). Increased sea surface temperatures above usual thresholds can result in coral bleaching which may lead to a benthic shift to stress tolerant coral species (22). In Kiribati, bleaching events have been recorded during extreme ENSO conditions in 2004–2005 and in 2009–2010, although the severity of coral bleaching was limited in 2009–2010 (23). Bleaching events may reduce structural complexity which provides critical habitat for fishes and protects against waves and storm surges—two critical ecosystem services (24). Urban areas may have combined impacts from climate change and human activity that could destabilize coral communities (23, 25). On outer islands with less human disturbance, coral communities maintain high diversity, but remain vulnerable to large-scale bleaching events and other local stressors (12).

### Economic Change in Kiribati

Pacific island economies have historically been dominated by subsistence and small-scale economic activities, with diets largely based on local foods, including seafood, root crops, starchy fruits, leafy greens, and coconut products (26). This is in part because the geographic isolation of island nations makes trade in perishable goods logistically challenging and costly. Yet, food imports, especially ultra-processed foods (e.g., sugar-sweetened beverages, ramen, spam), have increased throughout the Pacific, including in Kiribati (27). From 1961 to 2019, food imports to Kiribati outpaced population growth, with imports growing by 4 times (in terms of inflation-adjusted value; 27), while population grew by 2.8 times (28). Increasing imports is a result of—and reinforcing factor in—the shift toward a cash-based economy, urbanization, export promotion, financial integration, and development aid (29). These concurrent economic shifts are reflected in the percent of the population living in urban areas in Kiribati, which increased from <17% in 1961 to over 54% in 2019 (30), and increases in agricultural and fishery commodity exports, especially fish and coconut product exports (31). Collectively, these changes point toward a transition in market structure that is shifting the foods available and being consumed in Kiribati, with some I-Kiribati populations increasingly consuming more high fat and sugary processed foods (6).

### Water Quality

Marine pollution from land-based point sources (e.g., landfills, industrial waste, and sewage outfalls) and non-point sources (e.g., solid waste from humans and animals, heavy metals, wastewater, leaking septic tanks, pesticides, and fertilizers) are considered to be a primary threat to Kiribati coral reefs and thus, I-Kiribati health (12). Despite mercury and other heavy metals occurring in the environment naturally, anthropogenic sources such as urban runoff and pollution influence local levels and are considered a threat to coral reefs (32). Mercury is a ubiquitous metal in the environment that can have negative health effects, with seafood consumption a major source of human exposure due to its bioaccumulation across marine species food webs (33). While mercury exposure and levels of risk can vary due to human cultural practices (including eating), genetics, and ethnicity (34), even low doses can cause chronic issues (35). In Kiribati, there is limited mercury, heavy metal, and microbiological information [e.g., (36)], despite the very high consumption of seafood of I-Kiribati, and heavy dependence on reef-based resources. This study provides the first broad-scale assessment of mercury and heavy metals in plants, reef fish, and humans across multiple islands and reef habitats of Kiribati.

### Ciguatera

The incidence of ciguatera outbreaks, a food-borne illness produced from the dinoflagellate *Gambierdiscus spp*., can become more frequent as reefs degrade and algal growth increases (20, 37). *Gambierdiscus spp*. settle on the benthic substrate, such as algal turf or dead branching corals, and produce a ciguatoxin naturally (38, 39). The dinoflagellate is consumed passively as small-bodied reef fish graze resulting in the ciguatoxin bioaccumulating up the food chain following predation from larger bodied species (40). Thus, the incidence of ciguatera in reef fish catch can become more prevalent when reef degradation increases after a bleaching event or following nutrient input from anthropogenic sources (38, 41). For nations dependent on seafood, like Kiribati, ciguatera can have a profound impact on food security as there are scarce animal-source food alternatives if a ciguatera outbreak occurs (39). If a fish with high concentrations of ciguatoxin is consumed knowingly or not, neurological, cardiovascular, and gastrointestinal human health impacts can occur, with extreme cases resulting in fatality (38). In Kiribati, there are known high-risk areas where previous studies have found toxic concentrations (40), but little is known about the spatial variation, species of high-risk, and how long areas with known ciguatera outbreaks persist [but see (14, 40, 42)]. Our research will examine the prevalence, concentration, and distribution of ciguatera in marine food webs, and how this relates to dietary behaviors.

### Health Change in Kiribati

The impact of the above-mentioned economic, environmental, and climatic changes will pose threats to food availability, income generation, and local ecosystems, potentially affecting the nutritional status and associated disease burden of I-Kiribati. These health transitions have been well-articulated globally (43) and have particular importance in the Pacific, where declining infectious disease burdens are accompanied by increases in non-communicable diseases, or “diseases of modernization” (44). These transitions are largely driven by shifts in dietary patterns (5, 45, 46) and are often characterized by a reduction in undernourishment and a simultaneous rise in overnutrition (47).

## Methods/Design

### Study Aims

In this study, we aimed to (1) quantify the prevalence and variation of nutrition-related disease risk within and among islands; (2) characterize the external social-ecological risk factors that could shape disease status, including environmental health, food availability, and market access; (3) characterize the internal genetic and physiological risk factors that could shape disease status; and (4) analyze associations between these risk factors and nutrition-related disease at the individual, household, community, island, and national scale. It is also our hope to establish a baseline for future longitudinal research.

### Study Design and Setting

**Figure 1:**
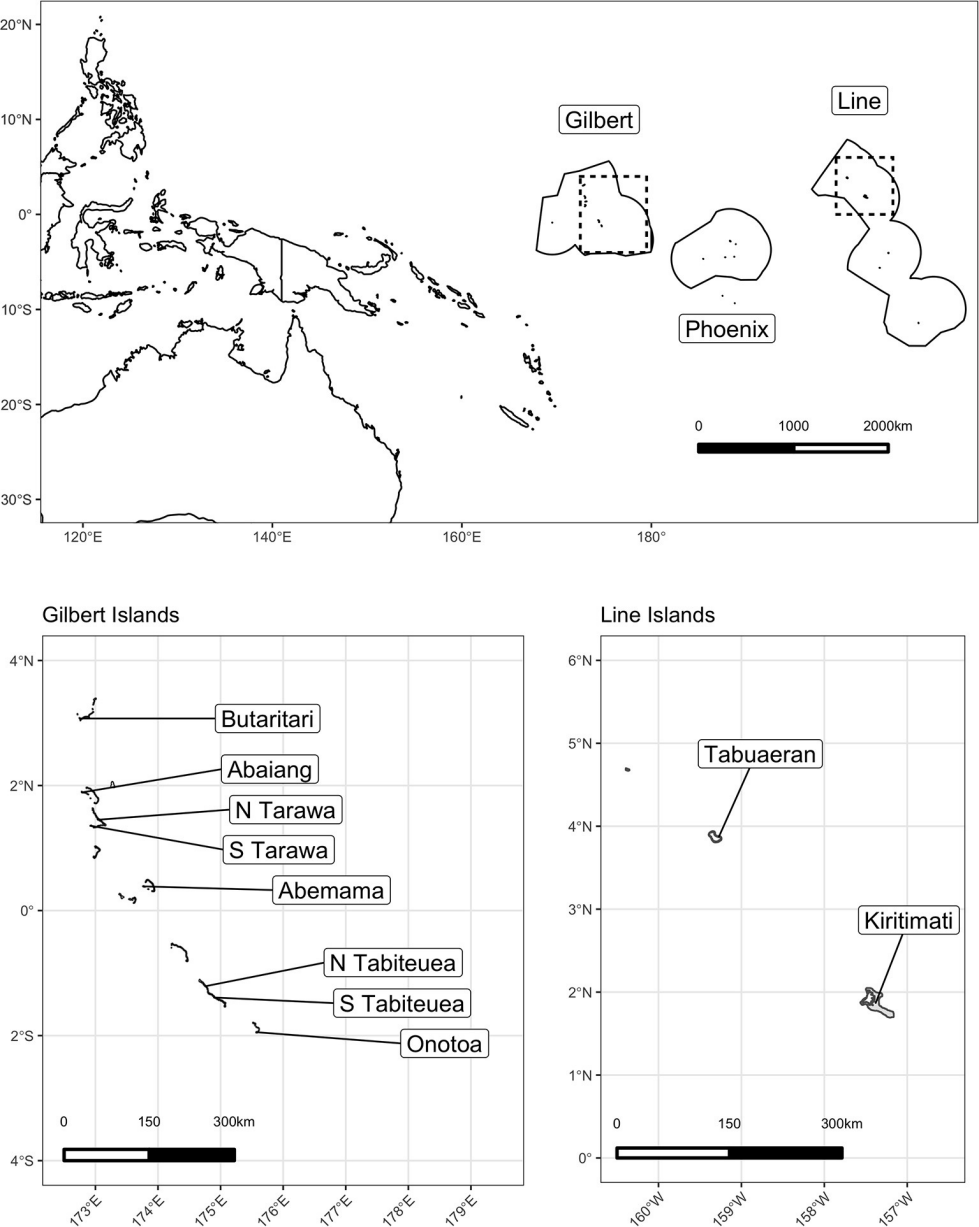
Map of the study area in the Republic of Kiribati.

We launched this study in the Republic of Kiribati, an independent nation of 33 low-lying islands in the central Pacific Ocean (Figure 1). The nation comprises three major island groups: the westernmost Gilbert Islands group, the sparsely inhabited central Phoenix Island group, and the easternmost Line Islands group. While Kiribati has a total land area of 811 km^2^ distributed throughout these three groups, it also encompasses ~3.55 million km^2^ of ocean area within their Exclusive Economic Zone, representing the largest ocean-to-land ratio in the world (48). Kiribati has a population of ~120,000 people, ~50% of which lives on the main Gilbert Island and capital of Kiribati, South Tarawa, and a per capita GDP of $1,636 USD, 9–16% of which emerges from fishing and related sectors (48–50).

Our mixed-method approach was embedded in a previously planned, nationally representative, observational cross-sectional study called the Household Income and Expenditure Survey (HIES) (50). The HIES is the equivalent to a Household Budget Survey, Household Expenditure and Consumption Survey, or a Living Standard Measurement Survey, which are generally conducted by all countries. The Pacific HIES is conducted by the Statistics Office of each national government, through technical and financial support from the Pacific Community (SPC), World Bank, the Food and Agriculture Organization of the United Nations and the International Labor Organization. The HIES collects information on household consumption and income, with the explicit purpose of estimating poverty and income distribution at national scales, and to rebase GDP and the basket of goods used in the calculation of the Consumer Price Index.

#### Creating the Integrated HIES

Our research integrates four separate research modules and is hereafter called the Integrated HIES. The first module was the original, standard HIES, which provides the demographic, social, and economic context to our broader research goals. The second module featured marine ecological research on the health and status of the coral reef system and associated ecological indicators. The third module focused on deepening the social, institutional, and economic dynamics that collectively shape local markets, and analyzed marine governance as a driver of interactions in this social-ecological system. The fourth module focused on the human health outcomes that arise from the social-ecological system dynamics, linking together the ecological, social, and economic modules.

By empirically researching each of these modules, we can adequately assess the nutritional ecology of diet-related diseases in Kiribati. Healthy marine ecosystems support healthy reef-based food systems, but do not guarantee positive health outcomes by themselves. A range of governance and market contexts mediate access to available food resources and are therefore necessary considerations for understanding nutrition security. These social and ecological domains are not, however, independent. Degraded coral ecosystems can drive changes in productive activities and market structures as communities are pushed into more cash-based livelihoods, while processes of globalization and urbanization can drive overutilization of marine resources. Such feedback processes can create a “social-ecological trap” that likely corresponds with differential nutrition outcomes (5). Notably, greater integration with global markets and the prevalence of cash-based economies is associated with higher consumption of highly processed, fatty, and sugary foods, facilitating the nutrition transition. Our sampling of the above four modules across a range of ecological health and market integration will allow us to identify how these dynamics may lead to different health outcomes.

#### Study Locations

Of the 21 islands that administered the HIES, we selected 10 islands to conduct the Integrated HIES, our focal research on social-ecological dynamics of coral reef-based food systems. We constrained our selection to only include islands whose geomorphology comprised a low-lying coral atoll with a ring-shaped coral rim partially enclosing a lagoon. Following that inclusion criteria to standardize our observations, islands were selected to represent a gradient of human pressure, coral reef health, governance, and market integration. Specifically, in order of prioritization: human population density, I-Kiribati national priority [see (11)], geographic location within the island group, spatial proximity to central markets and transit hubs, and availability of historical data were used as criteria for selection. Prior to finalizing the selection, a working group consisting of the Kiribati National Statistics Office (KNSO), Ministry of Health and Medical Services (MHMS), Ministry of Fisheries and Marine Resources Development (MFMRD), Pacific Community (SPC), American University (AU), Harvard T.H. Chan School of Public Health (HSPH), University of California Santa Barbara (UCSB), and University of California Santa Cruz (UCSC) met to discuss scheduling, transport, logistics, and budgetary restrictions. The final 10 islands were chosen (North to South) in both the Gilbert Islands (Butaritari, Abaiang, North Tarawa, South Tarawa, Abemama, Tabiteuea North, Tabiteuea South, and Onotoa) and the Line Islands (Tabuaeran and Kiritimati).

Within these 10 islands selected for focal research, three villages on each island were selected for human health and ecological research (a total of 30 villages). The three villages from each island were also selected to represent a gradient of human population size and market integration. Villages that require water transport to access the airport and/or central hub, which contains the Island Council and government buildings, were excluded. These included, but were not limited to, villages on coral cays, islets, or coral islands that were not connected by existing infrastructure. Villages additionally were selected to be geographically similar, where possible. Specifically, most Kiribati communities are located on the leeward (West) lagoon-facing side of the coral atoll arm adjacent to the back reef. Communities solely on the windward side (East), while rare, were not surveyed for focal research. Lastly, spatial distance between villages, discrete natural borders, historical data, and pre-existing governance research and relations, were considered. Coral reef and market research was conducted in areas within and adjacent to selected communities.

#### Training, Recruitment, and Enrollment

**Figure 2:**
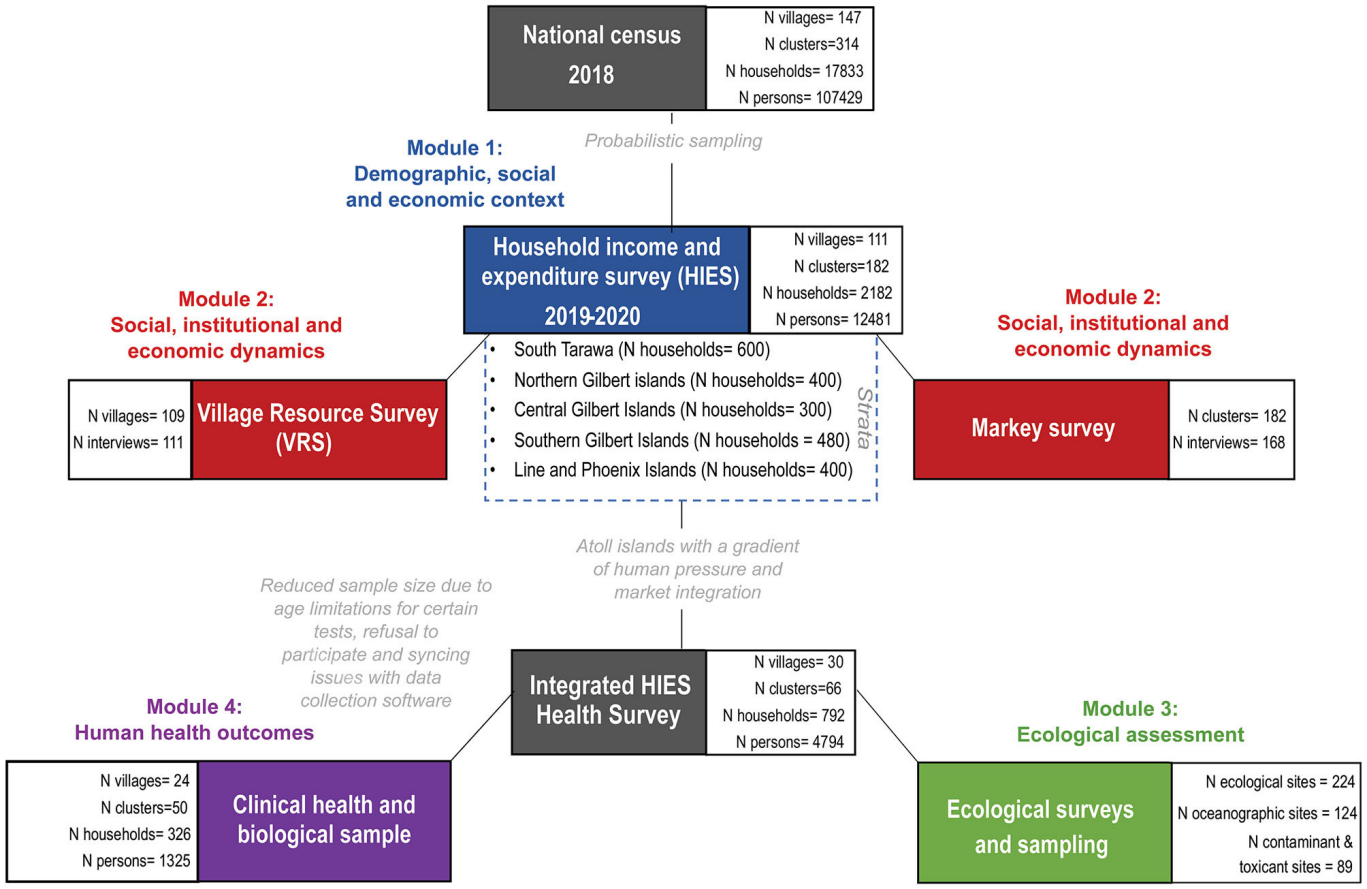
Consort figure detailing integrated study components.

KNSO in collaboration with SPC, conducted a national census in 2018 prior to the HIES, and, to the best of their knowledge, documented every person in Kiribati (Figure 2). Using this census as the sampling frame, and covering 21 of the total 23 inhabited islands of Kiribati, 182 clusters and 2,180 households were randomly sampled using probabilistic two-stage selection process for administering the HIES. The primary sampling unit (enumeration area) was based on probability proportion to size within each strata (South Tarawa, Northern, Central, Southern, and Line Islands) and households within selected enumeration areas were randomly selected. A total of 182 enumeration areas were selected in total (50 in South Tarawa, 33 in Northern, 25 in Central, 40 in Southern and 33 in Line Islands). Within each selected enumeration area, 18 households were randomly selected-−12 were targeted for interview with the other 6 selected as replacement households in case of non-response—using randomized steps to select individual households. A response rate of 100 percent was achieved, which included 12,481 individuals (4,287 in South Tarawa, 2,094 in Northern, 1,430 in Central, 2,397 in Southern and 2,273 in Line Islands).

The HIES enumerators were split into five teams to cover all targeted households across all of the surveyed islands. These five teams would work in parallel over the course of 1 year (May 2019–March 2020) to complete the entire sample, distributed across 21 islands in both the Gilbert and Line Island groups (Table 1). The HIES enumerator teams were all I-Kiribati citizens (predominantly women), recruited by KNSO and trained by experts from SPC.

**Table 1:** Study locations for the cross-sectional study in Kiribati with hypothesized reef health and market integration.

**Island name**	**Island group**	**Strata**	**Community name**	**Pop^**A**^**	**Number of HH (*n* sampled)^**B**^**	**Number of Ind. (*n* sampled)^**B**^**	**Rank reef health**	**Rank market integration**
Butaritari	Gilbert	Northern	Tabonuea	235	12	55	High	Low
Butaritari	Gilbert	Northern	Kuma	310	12	57	High	Medium
Butaritari	Gilbert	Northern	Ukiangang	655	24	133	High	High
Abaiang	Gilbert	Northern	Taburao	142	12	69	Med	Low
Abaiang	Gilbert	Northern	Tanimaiaki	312	12	53	Med	Medium
Abaiang	Gilbert	Northern	Tuarabu	548	12	70	Med	High
North Tarawa	Gilbert	Central	Tearinibai	441	12	59	Low	Low
North Tarawa	Gilbert	Central	Nooto	816	12	64	Low	Medium
North Tarawa	Gilbert	Central	Buota	1,647	24	138	Low	High
South Tarawa	Gilbert	Central	Nanikai	1,257	12	77	Low	Low
South Tarawa	Gilbert	Central	Bikenibeu	7,547	72	539	Low	Medium
South Tarawa	Gilbert	Central	Betio	18,565	168	1,273	Low	High
Abemama	Gilbert	Central	Tanimainiku	133	12	54	Med	Low
Abemama	Gilbert	Central	Tebanga	210	24	88	Med	Medium
Abemama	Gilbert	Central	Tabiang	618	24	98	Med	High
North Tabiteuea	Gilbert	Southern	Tauma	223	12	66	Med	Low
North Tabiteuea	Gilbert	Southern	Buota	436	12	71	Med	Medium
North Tabiteuea	Gilbert	Southern	Utiroa	774	24	118	Med	High
South Tabiteuea	Gilbert	Southern	Katabanga	82	12	76	High	Low
South Tabiteuea	Gilbert	Southern	Tewai	295	12	64	High	Medium
South Tabiteuea	Gilbert	Southern	Buariki	482	12	94	High	High
Onotoa	Gilbert	Southern	Buariki	208	12	55	High	Low
Onotoa	Gilbert	Southern	Aiaki	197	12	48	High	Medium
Onotoa	Gilbert	Southern	Temao	220	12	58	High	High
Tabuaeran	Line	Northern	Aramari	235	24	141	Med	Low
Tabuaeran	Line	Northern	Tereitannano	184	12	55	Med	Medium
Tabuaeran	Line	Northern	Tereitaki	370	12	62	Med	High
Kiritimati	Line	Central	Poland	404	24	161	Low	Low
Kiritimati	Line	Central	Banana	1,469	60	314	Low	Medium
Kiritimati	Line	Central	Tabwakea	3,573	96	584	Low	High

^a^*Kiribati 2020 census (https://kiribati.popgis.spc.int/)*.

*^b^Number of households (HH), and individuals (IND) recruited on each island*.

Prior to the beginning of the study, CG, WK, and JA traveled to South Tarawa to train MHMS nurses from every island in Kiribati to assist with the Integrated HIES health module research (Figure 2), particularly the measurement of hemoglobin and anthropometry. Five additional nurses were trained more comprehensively to conduct the focal clinical health research that took place in the aforementioned 10 islands. This training included specific protocols to ensure inter-nurse reliability for anthropometric measurements and detailed survey protocols to ask questions and enter information onto tablets. Additionally, JGE separately conducted training with four MFMRD Fisheries Officers in South Tarawa prior to the commencement of the ecological evaluation of coral reef health fieldwork. These field assistants learned the field, lab, and computing skills that were required to conduct sampling. Upon arrival to each island, the research team hosted capacity building meetings with the Island Council. The workshops covered (1) the project's research objectives, schedule, data collection and future reporting, and (2) the Island Council's research goals and aims, governance, fisheries objectives, historical information about the island, and areas of specific importance or interest, including any known ciguatera hotspots.

Of the 2,180 targeted households, we recruited 326 households into the focal clinical health research. Persons of both sexes and all ages were recruited into the research study, for a total of 1,305 individuals (Figure 2). There was no screening based on race or ethnicity. Households were defined as regularly cohabitating groups of individuals who pool resources and share meals. Subjects were offered no compensation for participating in interviews, or for providing clinical samples. However, subjects did receive the benefit of knowing the results of their point-of-care health tests. Individuals were recruited with a two-stage opt-out procedure. On the evening before the research would take place, a HIES enumerator and a nurse would visit the household selected by randomization. During that visit, the HIES enumerator would explain their research and the nurse would explain the health research, and provide them with the informed consent documents to review. The second stage of the opt-out procedure occurred the following morning when the nurse would arrive before breakfast to collect a signature for the informed consent document, conduct a brief survey, and to collect fasted biological samples. If a subject did not show up, it was assumed that the individual did not consent to the research. Surveys lasted ~15 min per person.

### Data Collection

#### Human Subjects Survey Data

##### Household Income and Expenditure Survey

The face-to-face survey, broken up into 40 rounds of collection, was implemented over a period of 10-months (May 2019–March 2020) in order to capture seasonal fluctuations in consumption, production and income. In addition to staff from KNSO, a total of 10 interviewers and 5 supervisors—divided into 5 teams—were employed to implement the survey. The survey questionnaire was developed in English using the World Bank Survey Solutions Computer Assisted Personal Interview (CAPI) software. The HIES questionnaire was divided into a series of modules (Supplementary Table S1).

Beyond the household-level food recall, there was also a module including a semi-quantitative food frequency questionnaire, where individual respondents within households were asked about the frequency of their consumption of 79 foods. They were also asked an open-ended question about whether they consumed any additional foods from five categories: cereals, seafood, fruit, vegetables, and snacks. These data were collected from all individuals aged 12 and older residing in households selected for the clinical health component of the research. Further information on the HIES, including the survey document, is publicly available (50).

##### Village Resource Survey

The Village Resource Survey comprised a maximum of 164 questions that provided context for the resources available within the village, recent environmental history, access to fishery benefits, and formal and informal rules around fishing and gleaning activities. The survey had seven sections. The first section detailed the characteristics of the interviewees, who were selected based on key informant criteria. The second section focused on property ownership and transfers. The third section included a series of questions about the proximity of the village to a range of public services, such as the nearest hospital, bank, and market. It also collected information about available transportation options. The fourth section asked about migratory work opportunities. The fifth section focused on fisheries assets, commonly targeted and consumed aquatic resources, recent changes in fisheries, including identification of recent natural hazards and coral bleaching events, and documentation of rules and customs related to fisheries. The sixth section detailed the availability of physical infrastructure, such as sewage and waste disposal. The final section focused on broad changes experienced in the village within the past 10 years.

Of the 147 villages in Kiribati, the HIES was implemented in 111 and it was intended that one VRS would be conducted in each village (i.e., VRS target was one interview to be conducted in 111 different villages). In total, 109 villages were included in the VRS with 111 interviews being completed (i.e., some villages were interviewed more than once). The VRS questionnaire was administered to village authorities. Respondents included elected village leaders (51), *unimane/unaine* (elders; 41), teachers (1), pastors (2), and others (5). The data were collected via interview of the HIES team supervisors using Survey Solutions Software.

##### Clinical Health Survey and Biological Sample Collection

The clinical health survey was conducted on a tablet using software from the Dharma Platform of BAO Systems. These questions were meant to complement the socio-economic, demographic, and health questions that were included—as standard—in the HIES. These questions focused on food taste preferences and health and vaccination histories. Targeted questions for reproductive-aged women included information on pregnancy, lactation, menarche, menopause, and birth control. The survey itself lasted only 10 min per individual. The survey, all physical health measurements, and all biological sample collection was conducted by one of five licensed nurses, who were employed by MHMS, overseen by HM and ET, and trained by CG, WK, and JA on specific research protocols. Because of internet and data syncing issues with the Dharma Platform, we lost a substantial amount of survey data.

#### Anthropometric Measures, Clinical Nutrition, and Disease Assessment

An I-Kiribati enumerator, as part of the HIES survey, recorded the following anthropometric measurements (Table 2): height/length and weight (all individuals); mid-upper arm circumference (children 5 years of age and under); and cranial circumference (children 2 years of age and under) using standardized WHO protocols (52). Additionally, a fingerprick was administered to all individuals <50 years of age, and hemoglobin and hematocrit measures collected through the use of a Hemochroma Plus device from Immunostics, Inc.

**Table 2:** Description of all survey data and instruments.

**Oceanography and microbiology assessment**	**Details**	**Sites**
Dietary intake	24-h and 1 week food recalls	All
Market dynamics	Product availability indicates whether the product is available all of the time, a couple days per week, a couple days per month, or seasonally	Village-Level
	Price represents the price of the item at the time of the survey	
	Units indicate the units in which the item is sold in standard units (e.g., pounds, kilograms, etc.) and nonstandard units (e.g., box, can, basket, etc.). The weight of items sold in nonstandard units were recorded for calculating weight standardization factors	
Socio-Economic status (HIES)	Household income and expenditure survey (see Supplementary Table S1)	Head of household, or other qualified respondent
VRS	Respondent Characteristics & Land Status—Key informant characteristics and perspective on key village features like leadership, participation, land tenure	Community key informants
	Transportation—Key informant perspective on transportation to key services within and beyond the village	Community key informants
	Industry—Key informant perspective on labor and occupations	Community key informants
	Fisheries—Key informant perspective on village trends in seafood catch, sale, and consumption, and comparisons to recent past and potential future	Community key informants
	Physical Infrastructure—Key informant perspective on infrastructure and services	Community key informants
	History & Development—Key informant perspective on comparisons between current village indicators and historical context	Community key informants
Fishing effort field surveys	Relative abundance of fishers by fishing method; maximum number (maxN) framework using line of sight estimates	Island-wide
**Clinical assessment**	**Details**	**Age group targeted**
Anthropometry	Height/length	All
	Weight	All
	Mid-Upper arm circumference	Children 5 and under
	Cranial circumference	Children 2 and under
Blood pressure	Measures systolic and diastolic blood pressure	Adults 12 and older
Glucose	Measures fasted circulating glucose	All individuals 12 and older
Metabolic disease *via* CardioCheck	Measures fasted total cholesterol, HDL cholesterol, and triglycerides	All individuals 12 and older
	Calculates LDL, TC/HDL ratio, LDL/HDL ratio, and non-HDL cholesterol	
Anemia	Hemoglobin and hematocrit	All individuals <50 years
Diabetes	Measures hemoglobin A1c	All individuals 12 and older
Fatty acid profiles	Analysis of dried blood cells by OmegaQuant. Provides readings of 23 different fatty acid profiles to understand contribution of seafood to nutrition	Male and female head of household and their oldest child
Mercury	Fingernail analysis for elemental mercury and methylmercury	All
Genetic markers	Evaluating genetic markers of physiological stress to better understand link between diet, anthropometry, and metabolic disease	All individuals 3 or older
Blood cell count	Evaluated from blood on microscope slides	All
**Ecological surveys**	**Details**	**Sites**
Marine community surveys	All diurnal fish—underwater visual census; belt transects	FR, LR, BR
	Highly mobile, rare, and flighty fish—underwater visual census; manta tows	MT
	All mobile and sessile invertebrates—underwater visual census; belt transects	FR, LR, BR
	Large-Bodied targeted invertebrates—underwater visual census; manta tows	MT
	Benthic habitat, relief, and complexity—uniform point contact; belt transects	FR, LR, BR
Infaunal surveys	All mobile, sessile, and infaunal invertebrates—sediment harvesting; soft infaunal quadrats and belt transects	SIQ
	Benthic habitat, relief, and complexity– sediment harvesting; soft infaunal quadrats and belt transects	SIQ
**Oceanography and microbiology assessment**	**Details**	**Sites**
Water samples	Salinity—salinity tester	FR, LR, BR, SIQ
	Conductivity—handheld conductivity meter	FR, LR, BR, SIQ
	Temperature—refractometer	FR, LR, BR, SIQ
	Chlorophyll A—filtered by glass micron membrane filter paper, stored, and preserved	FR, LR, BR, SIQ
	Nitrogen, Phosphorous, KH, Calcium—non-laboratory grade chemical	FR, LR, BR, SIQ
	Diatom collection—filtered by glass micron membrane filter paper, stored and preserved	FR, LR, BR, SIQ
	Microbiological samples—filtered by glass micron membrane filter paper, stored, and preserved	FR, LR, BR, SIQ
**Marine contaminants and toxicology**	**Details**	**Sites**
Heavy metal analysis	Macroalgae—*Halimeda opuntia* as a bioindicator of heavy metals	FR, LR
	Fish—liver and muscle tissue from groupers (Serranidae), snappers (Lutjanidae), parrotfish (Scaridae), and surgeonfish (Acanthuridae)	Island-wide; northern and southern sectors
Ciguatera analysis	Turf algae—microalgal community samples to assess presence and concentration of settled ciguatoxic dinoflagellates	FR, LR
	Fish—liver and muscle tissue from groupers (Serranidae), snappers (Lutjanidae), parrotfish (Scaridae), and surgeonfish (Acanthuridae)	Island-wide; northern and southern sectors

*Ecological habitats and sites: FR, fore reef; LR, lagoon reef; BR, back reefs; SIQ, soft-infaunal quadrats; and MT, manta tow*.

During the comprehensive clinical health survey that was administered on 10 islands, an I-Kiribati nurse collected a variety of measures and biological samples. Blood pressure was measured for all individuals over 12 years of age using an OMRON 10 series monitor. Measurements were taken after at least 10 min of sitting, and ensuring that individuals were as relaxed as possible. Subjects provided a maximum of three finger pricks of blood, using one 23-gauge lancet and two 30-gauge disposable lancets. Based on a subject's age, he/she received a different set of tests (Table 2). Using the two pricks with the 30-gauge lancets, the capillary whole blood was used to source samples for analyzing: (i) glucose levels with GenUltimate Blood Glucose Test Strips for use with the One Touch Ultra meter, (ii) total cholesterol, HDL cholesterol, and triglycerides using the CardioChek Plus Analyzer from PTS Diagnostics; (iii) hemoglobin A1c (a measure of diabetes) through the use of a A1cNow+ device from PTS Diagnostics; (iv) blood cell count through blood preserved on microscope slides; and (v) fatty acid profiles through OmegaQuant filter paper treated with HUFASave^TM^ (53). Additionally, dried blood spots were collected on Whatman filter paper FTA cards (2 spots per individual) for DNA extraction. Fingernails were also collected from each individual enrolled in the clinical health study, with the use of standard nail clippers, to assess the status of mercury and methylmercury contamination. Blood pressure, glucose, anemia, and diabetes results were provided at point-of-care, with referrals to local clinics or to the main hospitals as required. Filter papers were stored at −3°C in cool boxes until they could be stored for longer periods of time at the primary medical laboratory in South Tarawa at −18°C. In order to collect whole blood for gene expression profiling, we used a 23-gauge lancet to prick the thumb of our participants. Nurses collected ~10–15 drops of blood (~150 yl) in 1.5 yl cryotubes. The blood was stored in 500yl of 2x DNA/RNA shield (zymo cat # R1200). These samples were also stored at 4°C for up to a week and stored at −20°C thereafter.

#### Ecological Survey Data

To examine coral reef health we conducted ecological surveys across three distinct habitats: shallow outer fringing reefs or fore reefs (FR), lagoon reef (LR) pinnacles composed of reef-building corals, and subtidal soft sediment back reefs (BR). In addition to the primary FR, LR, and BR sites within each of the three habitat types, respectively, two additional sub-habitats were assessed using different methods: deeper fore reefs were surveyed by manta tows (MT) and nearshore low-tide exposed soft sediment back reefs were surveyed by soft-infaunal quadrats (SIQ). The location of sites within each habitat type were selected randomly to be adjacent to the focal communities and >1 km apart, where possible. For each island we designated 6 FR and 3 MT sites on the fore reef, 6 LR sites within the central lagoon, and 6 BR and 3 SIQ sites on the back reef (Figure 2). Selected sites within each survey type were consistent in depth, relief, aspect, and habitat structure. A subset of these surveys (3 FR, 3 LR, 3 BR, and 3 SIQ sites were randomly selected) included additional research on water quality.

##### Ecological Community Surveys

A team of ecologists (JGE, KKB, and ATeki) conducted marine community surveys to assess the species assemblage and functional health of I-Kiribati coral reefs. All diurnal fish species, non-cryptic invertebrates and the associated benthic habitats were quantified across the FR, LR, and BR sites by free-divers between 08:00 and 17:00 h. Observers were kept consistent to limit observer bias. For each site, three replicate 50 m transects were laid out using the belt transect method (*n* = 18 transects per habitat, per island; method described further in (24)) with >10 m spacing between transects. Transect placement was determined by habitat structure, depth (2–4 m), and heading.

##### Fish Surveys

Underwater visual census (UVC) was used to characterize the diurnal fish community assemblage. In order to avoid preferentially recording highly mobile and more bolder fish species and to limit diver effects (54), the fish observer (JGE) laid out the transect tape during the first “swim out” while simultaneously recording, identifying, sizing (nearest cm), and sexing all fish with a total length >10 cm across a survey width of 4 m (50 × 4 m benthic area per transect). On the second pass, less mobile, cryptic, small-bodied, and site-attached species were targeted with a more detailed examination of crevices across a swath of 2 m (50 × 2 m benthic area per transect). All individuals with a total length ≤ 10 cm were recorded, identified to the lowest recognizable taxon, sized (nearest cm), and sexed (when possible) as the observer more permanently fixed the transect. All fishes were recorded, including individuals in the water column within the defined benthic area (i.e., three dimensional surveys). When large schools or shoals of fish were present, species were binned by size class.

##### Invertebrate Surveys

After the completion of the UVC of fishes, a second observer (ATeki) entered the water to quantify the invertebrate composition and density along the fixed transects. For the assessment of all mobile and sessile invertebrates, a swath of 2 m was used (50 × 2 m benthic area per transect). All species were recorded to the lowest recognizable taxon if >50% of the individuals' body was inside the swath. To limit observer bias, “percent effort” was standardized. This included time spent searching, team assistance, conditions (i.e., visibility, surge, and swell), and the lack of additional visual tools (i.e., no underwater flashlight). In the instance of an overabundance of a particular organism a sub-sampling method was used in order to maintain percent effort. Sub-sampling requirements were met when >50 individuals of a species were recorded within one of the two 25 × 2 m transect sections (0–25, 25–50 m). When this occurred, the observer recorded the distance surveyed (in cm) and total count to extrapolate the density out for the remaining distance within that section.

##### Benthic Surveys

A benthic habitat assessment was conducted using a uniform point contact (UPC) method along the fixed transect (50 m). At each meter mark the observer recorded the identity of the primary space-holding organism, excluding epiphytes, or substrate beneath each point (*n* = 50). UPC cover categories consisted of macroalgae, turf, seagrass, sessile invertebrates (if a mobile invertebrate was landed on an adjacent point was used), non-biological substrate (e.g., sand coarseness, rubble, boulder), and corals [using definitions from (55)]. Low lying and unidentifiable masses of tightly packed turf-forming filamentous algae were recorded as turf algae [defined by Hay (56)]. All macroalgae and corals were recorded to species or lowest recognizable taxon by a consistent observer [KKB; using Kelley (57)]. For crustose coralline algae (CCA), encrusting calcareous algae, turf, or hard corals, growth form was classified as: branching, tabulate, digitate, plating, massive, submassive, and encrusting. When a live coral was landed on, the health of each colony was additionally assessed by recording percent bleached, presence and type of disease, and/or percent mortality when present. Bleaching scores were based on the 6-point color saturation scale on the CoralWatch Coral Health Chart *in situ* to minimize subjective assessment of bleaching state (58, 59). If bleaching was present, the coral was additionally characterized as either stressed or bleached and percent mortality of total coral structure was recorded.

In addition to point cover, the benthic observer recorded relief (*n* = 50) and structural complexity (*n* = 50) for each point. The change in vertical height between the shallowest and deepest substrate point within a 25 cm^2^ box was measured and recorded as relief. Relief was binned by depth: (1) 0–10 cm, (2) 10 cm−0.5 m, (3) 0.5–1.0 m, (4) 1.0–2.0 m, (5) 2.0–3.0 m, and (6) >3.0 m. Complexity characterized the porosity of the reef structure. For this study, an exponential scale of 1–>10 was used: (1) 1, (2) 2–3, (3) 4–6, (4) 7–10, and (5) >10. Similar to relief, complexity measurements were considered by vertical height, however, unlike relief measurements, complexity measurements were confined to the vertical plane of that specific point (i.e., not within a 25 cm^2^ box). For example, a point on the absolute bottom was scored a (1) where a highly complex coral, such as *Acropora* with >10 branches that crosses the vertical plane, was scored a (5).

##### Manta Tow Surveys

Fine-resolution reef community surveys using the belt transect method can occasionally underestimate rare species, large-bodied individuals, and highly mobile or flighty species (54). The manta tow technique, where a diver on breath-hold is towed behind a small boat, is a method that has been adapted to census these species and supplement belt transects. A primary advantage of the technique is that it enables an observer to census species of interest over large areas of reef benthos at speeds far greater than a free-swimming diver. We conducted surveys at 3 manta tow (MT) sites per island across a deeper aspect of the fore reef to account for underestimation of rare, large, and flighty species. Each MT site was established adjacent to a FR site, but was conducted at a deeper depth (6 m) and on a different day to limit observer bias (including boating disturbance). MT sites were additionally selected to have consistent reef substrate (*via* satellite imagery) and large channels or sand patches were avoided. At each site 6,300 m transects with a swath width of 10 m were surveyed (18 km^2^ observed per site) with >30 m spacing between transects. The tows were conducted by a consistent observer (JGE) at a constant heading and depth (±1 m due to tidal swings), in visibility >10 m, and at 3–4 km/h. A GPS was used to track speed, distance, and heading. Fish and invertebrate species of interest were recorded to the lowest taxonomic resolution, sized, and sexed.

##### Infaunal Surveys

While large-bodied reef-based invertebrates account for most of the I-Kiribati subsistence invertebrate catch (e.g., *Tridacna spp*.), a portion of fishing effort occurs on soft sediment habitat adjacent to the communities (7). Shellfish gathering, or gleaning, can fulfill nutritional needs when faced with fluctuations and seasonal inequalities in the availability of other resources. In order to quantitatively assess the abundance and diversity of species targeted by gleaning (animals that burrow and live beneath the substrate; e.g., the ark shell *Anadara maculosa* or *te bun* clams and *Trochus spp*.) we conducted soft infaunal quadrats (SIQ) on the soft sediment back reefs in areas exposed during low-tide [see Pakoa et al. (60) for more detailed methods]. SIQ sites were established 100–300 m inshore of the BR sites below the mean-high tide mark. Community assessments were made across a 40 m transect, laid parallel to shore, and replicated 3 times with >10 m spacing between transects. Observers randomly placed 4 25 cm^2^ quadrats within a 2 m swath along the transect for each 5 m segment (*n* = 32 per transect) and recorded the relief, complexity, substrate (mud, sand, rubble, etc.), and cover (seagrass, algae, sponges, epiphytes, etc.), when present. The observer then excavated, using traditional I-Kiribati hand and spoon harvesting methods (14), all sediment and organisms down to approximately 10 cm. The composition of the sediment and all visually identifiable species with >50% of the individuals' body inside the quadrat was recorded. During sediment sorting, “percent effort” (as described above) was not kept constant due to inconsistencies in the sediment. Instead, effort was considered complete when observers were 100% confident that all infaunal species within the sampling area were quantified.

##### Fishing Effort Field Surveys

To supplement the HIES and VRS fishing pressure estimates, *in situ* fishing effort surveys were conducted for all 10 focal islands. While the ecological field research was being conducted, observers (JGE, KKB, ATeki, and I-Kiribati boat drivers) visually quantified the amount of (1) motorboats, (2) paddle canoes (*waa n oo*), (3) sail canoes (*waa n ieie*), (4) shore-based net fishers, (5) shore-based spear fishers, (6) shore-based hook and line fishers, (7) gleaners, and (8) other efforts that were actively fishing and/or transiting to, between or after a confirmed fishing effort. The surveys were conducted using the maximum number (MaxN) framework to estimate relative abundance and were recorded twice daily; a.m. 8:00–12:59, and p.m. 13:00–18:00. Within the recording periods, all observers actively observed fishing effort within line of sight. MaxN is a commonly used method of estimating maximum relative abundance using video recordings or line of sight estimates when absolute observations are difficult to obtain (61). Thus, MaxN is a conservative method where the maximum number of samples observed at any one given time throughout the entirety of a distinct trial period is recorded (62). The method was designed to avoid the recurring counting of individuals that enter a field of view within a trial (63).

When a potential fishing effort was observed by line of sight during a trial period the observers watched for fishing behavior, fishing evidence and discussed the boats intentions with the local boat driver. A effort was not recorded when the observers were not certain, with reasonable doubt, that the effort was fishing based. When the method was not possible to decipher it was recorded as fishing other. However, most fishing effort was obvious, routine and verbally confirmed with the fishers when catch was observed. Influencing factors, such as the day of the week (limited fishing on Sunday), weather, wind, and tide, were recorded. Lastly, observer distance was noted as: a shore observation, lagoon near (associated village from departure/arrival location), lagoon far (outside the study sites for that village), leeward fore reef, windward fore reef or equal distance, and other to account for spatial differences in effort.

#### Oceanography and Microbiology Data

##### Oceanographic Measurements

To complement the ecological survey data and assess differences in oceanographic variables across the biogeographical and anthropogenic gradients, we collected 8 oceanographic measurements at 3 FR, 3 LR, 3 BR, and 3 SIQ sites per island. At each water sampling site, we measured (1) salinity, (2) conductivity, and (3) temperature using a handheld conductivity meter (YSI Model 30), salinity tester (Hach Pocket Pro) and a refractometer (Agriculture Solutions). All devices were calibrated prior to each measurement following the calibration specifications sheet provided by the manufacturer. Additionally, (4) chlorophyll A was sampled following the EPA Method 445.0 (64). Following EPA's guidelines, ocean water was filtered using a glass microfiber filter paper (Whatman 1823-025, 25 mm diameter) and then stored in a petri dish, wrapped in aluminum foil to prevent light damage, and frozen at −80°C. Lastly, (5) nitrogen, (6) alkalinity (KH), (7) calcium, and (8) phosphorus were tested *in situ* using a non-laboratory grade chemical kit for approximation and preliminary qualitative analysis.

##### Microbiological Samples

Three ocean water samples were collected at each site to assess site-level microbiology. For the first sample, ocean water was filtered through a glass micron membrane (Whatman 934-AH) to a volume of 150 mL of ocean water (~100 mL of cells) to identify *E. coli* and giardia and assess the general bacteriology and microbiology of the ocean water. The filter was transferred to a sterile tube and DNA/RNA Shield (Zymo Research) was added to ensure a 9:1 ratio (shield to ocean water cell solution). The filter was submerged and agitated to preserve all cells present on the filter surface. The above sampling and preservation process was repeated for the second sample, but with 9 mL of ocean water filtered through a glass micron membrane and preserved with 1 mL of DNA/RNA Shield. The second sample of higher ocean water volume supplements the first sample (of standardized volume) to ensure sufficient cells for analysis were captured if a site had a naturally low concentration of microbes. The third ocean water sample was collected to preserve and identify diatoms, dinoflagellates, and other primary producers. Ocean water was filtered again using a glass micron membrane, preserved in a 1% solution of 50% concentrated glutaraldehyde and shaken aggressively. All tubes were stored at −80°C.

#### Water Contaminants and Toxicants

Heavy metals and biotoxins in marine environments occur naturally and are known to be concentrated near coastal human populations due to nutrient pollution and runoff (65). Human health risks may present in traditional communities that depend on seafood as their primary animal-source food because of the bioaccumulation of mercury or presence of biotoxins such as ciguatera (42, 66). To link ecological, oceanographic, and microbial indicators to human health, we examined both heavy metals and ciguatera (known to be an issue in Kiribati). Algal samples and fish were collected by free-divers and fishers, respectively, across two of the three distinct reef habitats (3 LR and 3 FR per island) where hard coral substrate was present.

##### Halimeda

Quantifying concentrations of heavy metals in marine environments is challenging because trace metals (e.g., insoluble sulfur) can interfere with salt matrix refraction (51). Standard methods, such as inductively coupled plasma mass spectrometry (ICP-MS) with ISO 17294-2 protocol measurements can be used, but satisfactory performance results range from 41 to 86% with various metals (51). Instead, previous studies have used macroalgae as a bioindicator of heavy metals in saltwater (67–69). In the present study, *Halimeda opuntia*, a species of green macroalgae, was selected as a bioindicator of metals to establish a proxy of saltwater concentrations (70). Samples were collected underwater (>50 g per sample) along the ecological community survey transects with 2–5 m distance between samples, dry-weighed in the field (Hochoice milligram scale), and stored at −80°C.

##### Turf Algae

In order to explore the presence of ciguatera in microalgal communities, *via* benthic dinoflagellates of the genus *Gambierdiscus*, we sampled turf algae from recently degraded branching hard corals from the genus *Acropora*. Coral growth form, turf morphology, height and density [following (71)], temporal persistence and sediment load and composition (e.g., limited sand or silt) was standardized to the best of our ability *in situ* when selecting coral colonies for sampling. Turf algae was scraped, using a scalpel underwater, from the branch tips (>1 cm) of each colony along the ecological community survey transects with 2–5 m distance between coral colonies. Upon surfacing, the sample was divided into two tubes for later processing. DNA/RNA Shield was added to the first sample to ensure a 9:1 ratio (shield to turf algae) depending on the amount of turf collected. A 1% glutaraldehyde solution was added to the second sample to allow for microscopic imaging of the cells present. Turf algae samples were stored at −80°C.

##### Reef Fish

Reef fish were collected from both northern and southern sectors for each island to assess concentrations of heavy metals and ciguatoxin across geographical scales. Survey locations were selected during the initial capacity-building meetings with the Island Council as the most common fishing grounds. Discussions revolved around target catch (finfish) and reef habitat. To standardize fishing grounds across islands, only leeward fore reefs were sampled due to differences in island geomorphology (particularly lagoon reefs). To engage the community and ensure appropriate locations were sampled, we employed local fishers to catch reef fish for the study. Species were selected based on previously known I-Kiribati target species (14, 40). For each sector, we sampled 3 fish from each of the 4 families (family; example target species): groupers (Serranidae; *Cephalopholis argus, Epinephelus merra*), snappers (Lutjanidae; *Lutjanus bohar, L. gibbus*), parrotfish (Scaridae; *Chlorurus sordidus, Scarus frenatus*), and surgeonfish (Acanthuridae; *Ctenochaetus striatus, Acanthurus nigricans*). A total of at least 24 fish were sampled per island. While specific target species were requested and represented the majority of samples, other species from the same genera were occasionally substituted. Additionally, we opportunistically sampled other supplemental species, when present. If a ciguatera hotspot was mentioned by the Island Council (e.g., certain locations in Tabiteuea South), the area was sampled in addition to the two fishing sectors. For all reef fish, a geographic location within the sector was distinguished by having the fisher select a location using satellite maps. Fish were kept on ice or in a −17°C freezer, where possible, after catch, during transit, and for storage prior to dissections.

For each fish, liver and muscle tissue samples were taken for analysis of ciguatera and heavy metals, including mercury. After standard and total length were measured, the entire liver from the fish was removed and dissected into two 1 cm^3^ pieces. One sample was submerged in DNA/RNA Shield and stored for bacteriology, while the other standardized piece and remaining liver, if any, were stored in separate zip lock bags for metal analysis and future isotopic analysis, respectively. Scalpels were sterilized between tissue dissections with 90% isopropyl alcohol to avoid cross-contamination. The same procedure was followed for the white muscle tissue samples resulting in 6 samples per fish. Tissue samples were stored at −80°C. The animal study was reviewed and approved by University of California Santa Barbara IACUC.

### Data Analysis

The first module included general demographic and descriptive analyses at the individual, household, village, and island levels. These analyses utilized key basic indicators characteristic of HIES surveys, including variables such as geographic location, age, sex, poverty and food security status, and educational distributions, and provided foundational context for subsequent research modules on marine ecology (module 2), social, institutional, and economic dynamics (module 3), and human health outcomes (module 4). Further, these analyses provided key criteria for comparisons between islands and villages within our study (e.g., along gradients of population density), and across time scales (e.g., with past Kiribati HIES data).

#### Estimating Dietary and Nutrient Intake

The analytical methods applied to the 2019 HIES data are in line with the latest international best practices and regional guidance from the Pacific Statistics Methods Board (PSMB) on the construction of consumption aggregates. The consumption aggregate was finalized by the Statistics for Development Division of SPC, with input from the Food and Agricultural Organization of the United Nations and the World Bank.

##### Construction of the Consumption Expenditure Aggregate

*Food Consumption.* The monetary value of food consumption was attributed to the quantity consumed over the past 7 days for each food consumed by each food acquisition source (cash, own production, gifts, and exchange). The market survey was used to convert quantities of consumption expenditure that were reported in non-standard units of measurement to grams. Whole food acquisition was converted into edible quantities and nutrients using the Pacific Nutrient Database (72). Caloric availability was derived using the Atwater equation where 1 gram of protein = 4 calories, 1 gram of carbohydrate = 4 calories, 1 gram of fat = 9 calories, and 1 gram of pure alcohol = 7 calories (72). Only food consumed by the household was included, whether it was sourced from cash transactions, own-account production, gifting, or through exchange.

*Non-durable (and Non-food) and Semi-durable Consumption.* The consumption of non-food non-durable and semi-durable items was calculated as the annualized value of reported transactions for individual and household expenditures. Outliers were detected by product type and area in log-space using +/−2.5 × IQR and, where detected, locational medians were imputed.

*Durables.* Durables are items that are infrequently purchased by the household and have a long life (>1-year). Consumption of durables was estimated based on their use value.

*Intermediate Expenditure.* All expenditure related to productive activities and business were treated as intermediate expenditure and were not included in the consumption aggregate.

*Transfers.* All transfers (e.g., cash gifts to other households, or payments for fines) were excluded from the consumption aggregate.

*Imputed Rent.* The imputed rent component of the consumption aggregate was computed for owner-occupied housing using a predictive hedonic model, which was based on a range of variables including tenure, physical dwelling characteristics (number of rooms, building materials for walls, floor, roofing, water connection, flush toilet, electricity grid connection, fuel for cooking, and fuel for lighting) and location characteristics (province, urban/rural) characteristics. The model was based on rental expectations from the owner-occupying households because only 5 of the 2,182 households were renting, a sample deemed too small for an imputation model. For consistency across renting and non-renting households, the imputed rent from the model was used for all households, and actual rents were not used in the consumption aggregate. Deductions were made from the imputed rent for maintenance costs of owner occupiers.

*Dietary Intake Food Frequency.* Each of the 79 food groups were coded into their respective frequencies of consumption based on a provided serving size. All foods reported in “other” categories were translated, coded, and incorporated during the data cleaning and analysis stages. For each of the food groups, participants reported the frequency with which they consumed one serving based on the following options: (1) never/almost never; (2) once per month; (3) once per week; (4) three times per week; (5) daily; (6) more than daily; (7) more than three times per day.

#### Human Health Analyses

##### Clinical Health and Anthropometric Data Analyses

Anthropometric data were analyzed using standard WHO cutoffs to assess nutritional status. Children under 5 were assessed as underweight (weight-for-age z-score < -2), being stunted [height-for-age z-score (HAZ) < -2], wasted (weight-for-height z-score < -2) and overweight (weight-for-height z-score > 2) based on the WHO Child Growth Standards (52). Mid-upper arm circumference (MUAC) was used to assess severe acute malnutrition in children based on a MUAC <11.5 cm in children under 5, and moderate acute malnutrition based for a MUAC ≥ 11.5 cm and <12.5 cm. For children <24 months, a head-circumference-for-age z-score < -2 was classified as a case of microcephaly. In children ages 5–19, overweight was classified based on a body-mass-index-for-age z-score (BMIZ) > 1, and obesity was classified as a BMIZ > 2. In adults ages 20 and above, body mass index (BMI) was used to classify nutritional status in the following way: underweight, BMI <18.5; 18.5–24.9, normal weight; 25.0–29.9, overweight; 30.0–34.9, obesity class I; 35.0–39.9, obesity class II; and >40, obesity class III. Observations were considered to be outliers if their WAZ or HAZ were > |6| or their head circumference z-score or WLZ were > |5|.

Anemia status was classified into non-anemia or mild, moderate, or severe anemia based on hemoglobin levels (g/dL) with respect to age and pregnancy status per the WHO recommendations (73). In children 6–59 months of age, anemia was classified as follows: mild, 10.0–10.9 g/dL; moderate, 7.0–9.9; and severe, <7.0. In children 5–11 years of age: mild, 11.0–11.4 g/dL; moderate, 8.0–10.9; and severe, <8.0. In children ages 12–14 and non-pregnant women over age 15: mild, 11.0–11.9 mg/dL; moderate, 8.0–10.9; and severe, <8.0. In pregnant women: mild anemia, 10.0–10.9 g/dL; moderate, 7.0–9.9; and severe, <7.0. Lastly, men ages 15 and above were classified as having anemia as follows: mild, 11.0–12.9 mg/dL; moderate, 8.0–10.9; and severe, <8.0. Non-anemia was designated in the absence of anemia per the classifications of each group.

Blood pressure was considered to be: normal if systolic <120 mmHg and diastolic <80 mmHg; elevated if systolic was ≥120 and ≤ 129 mmHg and diastolic <80 mmHg; Stage 1 hypertension if systolic was ≥130 and ≤ 139 mmHg or diastolic was ≥80 and ≤ 89 mmHg; Stage 2 if systolic ≥140 and ≤ 180 mmHg or diastolic ≥90 mmHg; and Hypertensive Crisis if systolic > 180 mmHg and/or diastolic >120 mmHg (74).

Metabolic syndrome, and its constituent measures, all followed from American Heart Association guidance (75). In individuals over age 12, fasting plasma glucose levels ≥180 mg/dL were considered hyperglycemic; 70–180 mg/dL normal, and ≤ 70 mg/dL hypoglycemic. An individual was considered to be pre-diabetic if their hemoglobin A1c (HbA1c) levels were between 5.7 and 6.5%; diabetic if their HbA1c ≥ 6.5%; and non-diabetic if their HbA1c <5.7%. Cholesterol levels were considered normal if total cholesterol <200 mg/dL, HDL > 40 mg/dL, and LDL <130 mg/dL. Cholesterol levels were considered at-risk if total cholesterol ≥200 or HDL ≤ 40 or LDL ≥ 130. Triglycerides were assessed as follows: <150 mg/dL as low-normal; ≥100 and <150 mg/dL as high-normal; ≥150 and <200 mg/dL as borderline hypertriglyceridemia; ≥ 200 and <500 as moderate hypertriglyceridemia; and ≥500 mg/dL as severe hypertriglyceridemia. Note that the CardioChek Plus cannot read triglyceride values below 50 mg/dL, therefore neither triglyceride nor LDL cholesterol values were recorded for these individuals. LDL cholesterol is estimated as a function of triglyceride, HDL cholesterol, and total cholesterol levels using the Friedewald Equation (LDL cholesterol = total cholesterol—HDL cholesterol—triglycerides/5) (76).

##### Fatty Acid Profiles

All OmegaQuant filter papers, treated with HUFASave^TM^, were sent to OmegaQuant laboratories for fatty acid analysis following established protocols (53). Their analyses produce 24 fatty acid profiles, including eicosapentaenoic acid (EPA), docosahexaenoic acid (DHA), and an overall omega-3 index, which has been associated with various forms of morbidity and mortality (53).

##### Genetic and Immunological Analyses

DNA was extracted from dried blood spots using Zymo Quick-DNA extraction Kits. Participants will eventually be genotyped using Illumina's Infinium Global Screening Array v1.0 (GSA) from Illumina (77), which includes 642,824 SNPs. Genotypic calls and CNV analysis will be performed using the Genome Studio 2.0 (Illumina Inc. SD, California). We will exclude participants if (i) genotyping fails at more than 5% of SNPs, and/or (ii) if their heterozygosity rate is >3 standard deviations from the cohort mean. We will also exclude rare alleles (minor allele frequency (MAF) <0.01) and those that are not in Hardy–Weinberg Equilibrium (*p* < 1 × 10–6). LD will be calculated using *r*^2^ implemented in PLINK (78) and removing one SNP of a pair each time *r*^2^ > 0.6.

Two blood smears (Peripheral Smear) were collected for each participant to calculate blood cell counts. Following fixation, each slide was evaluated for differential white blood cell count (WBC). This count was done manually using a microscope and a cell counter for neutrophils, lymphocytes, monocytes, eosinophils, and basophils. WBC percentages were standardized for each WBC. Normal ranges in adults are: neutrophils (55–70%), lymphocytes (20–40%), monocytes (2–8%), eosinophils (1–4%), and basophils (0.5–1%) of total white blood cells (79). We also evaluated each participant for RBC irregularities (e.g., anisocytosis—indicative of anemia; poikilocytosis and anisopoikilocytosis), the presence of abnormal cells (e.g., presence of immature white blood cells such as blasts, indicative of leukemia, or serious bone marrow disease) and infectious agents. Note that a variety of diseases and conditions can affect the relative number of WBC, and we primarily intend to use variation in WBC as a covariate in downstream analysis (e.g., blood gene expression profiling).

#### Ecological Analysis

The second module featured marine ecological research on the health and status of the coral reef system. To examine how coral reef health influences human health, the ecological research module focused not only on species with key functional roles, but also on species and ecosystems significant to I-Kiribati fisheries, food security, and cultural importance. Ten ecological indicators (EI) across multiple dimensions were used to assess ecosystem health, capture natural variation, and incorporate interactions between species and the environment. Variability in the structural composition and geomorphology of coral reef atolls, as described above, constitutes distinct ecosystems, or habitats, with different species compositions and oceanographic conditions. The back reef, where SIQ infaunal survey data was collected, hosts a different, but important, assemblage of species than those found on the atoll's lagoon or fore reefs. Thus, analysis was focused on the status of different species assemblages across all habitat types.

We use (EI 1) abundance, (EI 2) richness, and (EI 3) size structure data of frequently harvested and functionally important fish and invertebrate species from the community surveys as primary ecological indicators. For example, species such as the humpback red snapper *Lutjanus gibbus* or green humphead parrotfish *Bolbometopon muricatum*, respectively, can offer insights into the relative health of the lagoon reefs or fore reefs when using these three indicators. With the community survey data, we then assigned fishes to (EI 4) broad trophic categories and converted counts and total lengths to (EI 5) biomass density using trophic classifications and length-weight conversion compiled by the Ecosystem Science Division of NOAA's Pacific Islands Fisheries Science Center from FishBase (80). We hypothesized that degraded and overfished reefs would have lower biomass density of trophic groups targeted by fishers, such as large predators. Fish biomass, coupled with trophic structure, has been used globally as a primary way to describe coral reef function. However, while trophic structure depicts the entire systems' connectivity of direct and indirect feeding relationships, species within trophic groups operate in different functional roles that influence the ecosystem's health, resilience, or resistance to impacts or shock. Thus, we further assessed the health of the species assemblage and ecosystem by examining (EI 6) the functional structure (81) and (EI 7) overall biodiversity [measured by using functional structure and species richness; see (82)]. For example, reefs that have great biomass at intermediate consumer levels, which can be described as “middle-drive” systems are functionally different from reefs with trophic structures that appear top-heavy (80).

While the community, manta tow, and infaunal surveys provided a way to evaluate the frequently harvested and functionally important fish and invertebrate species, the benthic surveys also contributed supplementary ecological indicators of coral reef health. The transition from a coral-dominated reef to a degraded, macroalgal-dominated reef is the most common way to assess coral reef functional health when comprehensive benthic data is available (83). Thus, the (EI 8) percent substrate cover and proportion of scleractinian framework-building corals vs. macroalgae and/or turf was used to determine the level of degradation across sites and islands. Similar to EI 6 for fishes, we then assessed the (EI 9) functional diversity of the coral assemblage using the life history categories proposed in Darling et al. (84): competitive, stress tolerant, generalist, or weedy. The categorizations were developed and synthesized from a trait-based approach with species characteristics of colony morphology, growth, calcification and reproduction (https://coraltraits.org). Analyzing the functional classifications of corals, when assessed proportionally, can indicate the frequency and intensity of thermal disturbances, anthropogenic impacts, and together, the levels of historic degradation. Shifts in dominant traits from competitive habitat specialists to weedy generalists also can be attributed to the structural complexity of the habitat (24). As coral reefs degrade, the cover of total live coral and branching corals decreases and structural complexity is lost, which decreases the resilience of coral reefs to disturbances (85). Thus, because structural complexity is an integral component of the coral reef ecosystem, (EI 10) the relief and complexity were analyzed as the final benthic ecological indicator.

#### Oceanographic Analysis

In addition to the ten ecological indicators, oceanographic indicators also can modify ecosystem health. Eight biophysical drivers were considered to evaluate the quality of the ocean water and further describe current reef conditions. We measured (OI 1) salinity and (OI 2) conductivity to indicate the concentrations of salt ions and dissolved solids in water, respectively. Temperature data (OI 3), both current and historic, was used to monitor current conditions, which can be a precursor to harmful algal blooms and coral reef degradation. For *in situ* data, (OI 4) chlorophyll A was used to measure primary productivity as an indicator of runoff. We coupled the chlorophyll A data with nitrogen, alkalinity (KH), calcium and phosphorus within the analysis to triangulate water quality due to the biochemical properties of ocean water (unlike freshwater tests). Due to the variability of ocean water composition, reported concentrations may not always accurately represent long-term values, particularly when comparing offshore and in-shore environments (86, 87). Nitrogen (OI 5) measurements were used to quantify excess bacterial decomposition in water, particularly in locations close to shore (e.g., SIQ sites) and near densely populated areas (highly developed communities). The combination of (OI 6) calcium and (OI 7) KH highlighted approximate levels of dissolved calcium present in the reefs, as well as the stability of those calcium ions. KH was specifically used to demonstrate the potential buffering capacity across the study sites. Lastly, (OI 8) phosphorus was measured, as an overabundance may signal a eutrophication event and the potential for harmful algal blooms, which may be associated with ciguatera outbreaks (88).

By using the 8 oceanographic measurements and historical data on climate impacts (i.e., NOAA Coral Reef Watch: degree heating weeks), we can produce maps of spatially explicit climatic influences. Together, these variables provide a baseline environmental gradient to evaluate anthropogenic impacts on coral reefs. Eventually, we will evaluate these biophysical drivers in conjunction with heavy metals, diatom species identification, and ciguatoxin analysis to examine associations with human health.

#### Water Contaminant and Toxicant Analysis

##### Metal Analysis Preparation

To prepare samples from *Halimeda opuntia* and fish for analysis, they were removed from a deep freezer (−80°C), lyophilized (MechaTech Systems, LyoDry Compact), and transferred to sterile tubes (VWR Centrifuge Tube, 76204-404, Batch: 190717060 and 190609058). Samples were ground using a metal spatula sterilized using 10% Hydrochloric acid (J.T. Baker, Hydrochloric Acid 36.5–38.0%, Baker Instra-Analyzed Reagent, 9530-33, Batch No: 000024173 diluted with deionized water). Tissues were weighed for dry-weight before Thermo Quant'X EDXRF (XRF) and mercury analysis (Mettler-Toledo AG XPE205DR).

##### Total Mercury Analysis

Ground samples were analyzed at Harvard University for total mercury concentration (T-Hg) on a direct mercury analyzer (DMA) (NIC MA-3000 Mercury Analyzer and MA3 Win software) following US EPA Method 7473 (US EPA 1998). This process involves measurement by thermal decomposition, reduction, amalgamation, and atomic absorption spectrometry. A quality control and assurance series was run every 12 samples. This series included the following: blanks (2), deionized water purge (1), dry purge (1), Apple Leaves 1515 (1) (NIST Standard Reference Material (SRM), US Department of Commerce National Institute of Standards and Technology, Gaither, MD 20899), MESS-4 SRM (1) (Marine Sediment SRM, National Research Council Canada, Lot G 4169010, Serial CC 567765) to verify recovery rates and sample integrity. Calibration curves were set using *R*^2^ > 0.99 and SRM recovery was 87–102% confirming the accuracy of measurements. Amalgamator and catalyst tubes were replaced as needed due to excessive calcium carbonate deposition from the tissues.

To prepare human fingernail samples, they were cleaned with a 1% triton deionized (DI) water solution, sonicated for 30 min, vortexed, and soaked for an additional 2 days (89). This process of sonification and rinsing was repeated three more times, including an acetone rinse, and nails were then freeze-dried overnight. Total mercury (Hg) concentrations were measured with a Nippon MA-3000 mercury analyzer (U.S. EPA 1998) along with a certified reference material (European Reference Materials (ERM-DB001), human hair).

##### Heavy Metal Analysis

To test the concentration of heavy metals present, samples were analyzed using inductively coupled plasma mass spectrometry (Thermo Scientific, iCAP Q) following EPA Method 6020B (U.S. EPA 2014). This process involves ~100 mg of dried samples digested at 95°C for 6 h using a 4 mL nitric acid and hydrogen peroxide solution (3:1, respectively, J.T. Baker, Instra-Analyzed Reagent, Batch No: 0000221802). Post digestion, samples were diluted to 30 mL using deionized water and measured on ICP-MS. Calibration of ICP-MS was conducted using Multi-Element Solution 2 (Spex CertiPrep, USA). Average recovery rates for calibration standards and trace metals in water SRM (NIST 1643f) analyzed as samples were generally in the range of 90–110%, supporting measurement accuracy. These results allowed for comparison of mercury values between two machines. All samples were then tested for heavy metals using X-ray Fluorescence, which is a faster analysis process.

We used a Thermo ARL Quant'X EDXRF (XRF; Thermo Fisher Billerica, MA) for non-destructive analysis of a suite of elements. The system was run for 30 min live time for each sample with x-ray settings of 50 kVp and 1 mA using a silver filter and anode. Samples were measured in 30 mm diameter, polyethylene XRF sample cups with a 2.5 um mylar support film. XRF was optimized for sampling over a 20 mm^2^ area to a depth of approximately 0.5 cm. Samples would rotate during measurement to ensure a full homogenous measure of the cup and sample. XRF spectra was analyzed using in-house peak fitting methods calibrated against standards with known composition (89).

##### Water Microbiological Analyses

Water samples will be analyzed to explore the presence of various human relevant diseases. DNA/RNA solutions can be later used for extraction and DNA sequencing to screen for bacteriology analysis which can detect cryptosporidium, fecal coliforms, giardia, and general bacterial presence. We will use a combination of 16S rRNA and shotgun metagenomics sequencing to identify variations in community composition that may correlate with adverse health outcomes.

##### Ciguatera Analysis

Reef fish samples (liver and white muscle tissue) and turf algal samples were shipped to the National Oceanic and Atmospheric Administration (NOAA) to analyze the presence of ciguatoxin (CTX) using their fluorescent receptor binding assay protocol (90). This process involves a fluorescence-based receptor binding assay [RBA_(F)_], based on competition binding between CTX and fluorescent-labeled brevetoxin-2 with voltage-gated sodium channel receptors. The analysis detects the presence or absence of ciguatoxin, toxicity levels, and the percent binding equivalents.

#### Social, Institutional, and Economic Analyses

To examine how informal and formal governance structures mediate access to reef fish and their resource benefits, the governance research module combined data collected from both the HIES and the VRS surveys. As a representative sample of I-Kiribati households, the HIES provided standardized quantitative data on: household knowledge of and adherence to formal (e.g., Government of Kiribati) and informal (e.g., traditional, religious) fisheries rules and customs; inter- and intra-household dynamics of reef fishing and reef food access; prevalence and diversity of practices such as catch sharing and gifting; and how individual households with various characteristics utilize reef resources and access reef foods. Additionally, as a key informant survey of local village representatives, the VRS provided additional insights into: the specific rights, rules, and decision-making procedures applicable within a village; criteria for participation in various forms of village and fishery institutions, capital and assets available within the village or held in common, and historical context for the data provided by households through the HIES.

To examine how markets and trade mediate access to reef fish and their resource benefits, the market survey provided data on product availability, frequency of availability and price by village. We construct a product diversity score based on the market data and pair this with VRS data on proximity and travel time to other market centers. Together, these variables serve as an indicator of the market integration with formal markets in each village. We compare the prevalence of processed and high fat/high sugar foods available in the formal markets to the local produce and animal products sold within the village to measure the degree of nutrition transition of the supply.

### Ethics Approval and Consent to Participate

The HIES was implemented by the Kiribati National Statistics Office in accordance to the 1974 Statistics Ordinance, which assigns authority to the Republic Statistician to collect and compile information relating to, among other areas, household expenditure, health and fishing. For the clinical health research, all households were recruited and enrolled, and each individual consented or assented, following our IRB approved study (Protocol #18-0967, Committee on the Use of Human Subjects, Office of Human Research Administration at the Harvard T.H. Chan School of Public Health). The study was also approved by the Ministry of Health and Medical Services in Kiribati. All point-of-care health results (anemia, diabetes, hypertension, etc.) were provided by our team's health professionals to the study subjects including access to free treatment and referrals to local clinics.

## Interim Results

We found a moderate prevalence of stunting (23.6%), wasting (6.9%), and underweight (10.1%) throughout the study population (Table 3). Yet, 78.8% of the population is either overweight (32.4%) or obese (46.4%), indicating an environment of chronic overnutrition whereby individuals are consuming too much dietary energy. There is a moderate prevalence of anemia in both reproductive-aged women (21.9%) and children under 5 years of age (34.2%). The population is heavily left-skewed with more than 40% of the population being <18 years of age, indicating rapid population growth in this region. Laboratory analyses are still in progress; baseline HIES point-of-care results are shown in Table 3.

**Table 3:** Demographic and disease status summary statistics of the enrolled HIES study population^a^.

	**Overall**	**Gilbert**	**Line**	**High development**	**Low development**
*n*	12,351	10,080	2,271	6,222	6,129
**Age group**					
0–5 years	14.9	15.0	12.7	15.5	13.7
6–11 years	14.3	14.3	14.6	13.5	15.7
12–17 years	10.9	10.6	14.5	10.1	12.4
18–49 years	45.5	45.7	43.3	47.8	41.5
50+ years	14.4	14.4	14.9	13.0	16.8
Respondent is female (%)	49.3	49.5	46.2	49.6	48.7
% Pregnant, women ages 15–49	5.5	5.3	8.8	5.0	6.6
**Anemia status, women ages 15–49**					
Mild	9.7	9.9	7.3	8.8	11.3
Moderate	9.5	9.7	6.1	9.1	10.2
Severe	1.7	1.8	0.6	1.3	2.4
Non-anemic	79.2	78.6	85.9	80.8	76.1
**Anemia status, children** ** <5**					
Mild	14.2	14.2	15.2	12.8	16.9
Moderate	17.3	16.9	21.9	13.9	23.7
Severe	2.7	2.8	1.3	1.1	5.9
Non-anemic	65.8	66.1	61.7	72.2	53.4
**Anthropometry, children** ** <5**					
**Stunted (%)**					
Moderate stunting	16.1	16.0	17.1	16.5	15.4
Severe stunting	7.5	7.2	10.7	6.9	8.6
Not stunted	76.5	76.8	72.2	76.7	76.0
**Wasted (%)**					
Moderate wasting	4.1	4.2	2.2	3.7	4.9
Severe wasting	2.8	2.9	1.2	2.9	2.5
Not wasted	93.1	92.9	96.6	93.4	92.7
**Underweight (%)**					
Moderate underweight	7.1	7.1	6.4	7.3	6.6
Severe underweight	3.0	3.1	2.2	2.3	4.3
Not underweight	89.9	89.8	91.4	90.4	89.0
**BMI of adults 18+**					
Underweight	0.9	0.9	0.6	0.7	1.2
Normal weight	20.4	20.6	18.1	18.1	24.5
Overweight	32.4	32.5	30.3	32.5	32.1
Obesity class I	26.2	26.1	27.9	27.0	24.9
Obesity class II	13.9	13.8	14.8	15.0	11.9
Obesity class III	6.3	6.1	8.2	6.8	5.4

^a^*The sample sizes for the variables in this table (due to missing data and data subsetting) are as follows: age group and sex = 1,351; pregnancy = 2,353; anemia status of women 15–50 = 2,429; anemia status of children < 5 = 1,407; anthropometry of children < 5 = 1,392; BMI of adults 18+ = 6,201. BMI, Body mass index. Child anthropometry excludes suspected data errors with weight-for-age z-scores >6 or < -6; length/height-for-age z-scores >6 or < -6, or weight-for-length/height z-scores >5 or < -5 (a total of 71 children). Moderate wasting/underweight/stunting is classified as a z-score < -2, and severe wasting/underweight/stunting is classified as a z-score < -3. Adult BMI was calculated as kg/m^2^ and excluded for a BMI > 80, or a height < 111.8 or >228.6 cm; or a weight < 24.9 or >453.6 kg (a total of 56 adults). Anemia was calculated and categorized based on the WHO guidelines accounting for age and pregnancy status (73)*.

## Discussion

The main strength of this cross-sectional study is that the HIES modules are nationally representative and include both sexes and all ages of I-Kiribati society of the Gilbert and Line island groups. Moreover, a subset of these islands (ten in total) became part of what the Pacific Community is calling the Integrated HIES that connects ecological and human health data collection to the ongoing social, demographic, and economic data collection of the HIES. The integrated HIES becomes a more powerful study design to understand the underlying ecological drivers of social and economic patterns that can lead to various human health sequelae [e.g., (5)].

Our initial results paint an unfortunately typical picture of health status in the Pacific with moderate prevalences of undernutrition and anemia and an extremely high prevalence of obesity. Furthermore, given the vulnerability of this region to climate change, this population is characteristic of what is termed a global syndemic connecting undernutrition, obesity, and climate change (91). As this syndemic creates inherent connections among climate, environment, and human health, it is essential that the Integrated HIES continues to collect data and monitor populations so that governments and policymakers can learn and adapt to changing conditions.

The primary limitation of this study design is that we are establishing a baseline for future work, and thus it does not yet include longitudinal data. Future longitudinal data will allow us to understand the ways in which changing environments, and changing climates, may shape food systems and dietary intake patterns, thus influencing health outcomes such as obesity, hypertension, and diabetes.

## Ethics Statement

The studies involving human participants were reviewed and approved by Harvard T.H. Chan School of Public Health Office of Regulatory Affairs and Research Compliance. Written informed consent to participate in this study was provided by the participants' legal guardian/next of kin. The animal study was reviewed and approved by University of California Santa Barbara IACUC.

## Author Contributions

The study was designed by CG, JA, JE, JG, KS, MS, DM, KN, and AT. CG, JA, and KN led the human health modules of the study. JE led the ecology module of the study. MS, KS, and JG led the VRS and market components of the study. MS and AT led the HIES implementation. KS led the social and governance research modules. HB, PB, AS, ES, and CG led the heavy metal analyses. HB, JE, and CG led the ciguatera study design. HM, RT, ET, and KN led the local management of health staff and research sample collection. KB, HB, and ATeki assisted with the ecology module and WK assisted with the human health module. JK assisted with the data collection infrastructure for the health research. KG, SP, NN, MS and JM cleaned, coded, and analyzed data collected from the research. CG, JE, JG, KS, MS, and HB drafted the manuscript. All authors have edited and approved of thefinal manuscript.

## Funding

We are grateful for the financial support of the National Science Foundation (CNH 1826668), in-kind support from the Dharma Platform of BAO Systems, and the leveraged funding from the Pacific Community (SPC) to collaborate with the Government of Kiribati on their Household Income and Expenditure Survey (HIES). MS was funded by the Australian Government through Australian Center for International Agricultural Research (FIS/2018/155).

## Conflict of Interest

WK finished his contribution to this article prior to leaving the Harvard T.H. Chan School of Public Health for a position at Impossible Foods. JK was employed by the BAO Systems. The remaining authors declare that the research was conducted in the absence of any commercial or financial relationships that could be construed as a potential conflict of interest.

## Publisher's Note

All claims expressed in this article are solely those of the authors and do not necessarily represent those of their affiliated organizations, or those of the publisher, the editors and the reviewers. Any product that may be evaluated in this article, or claim that may be made by its manufacturer, is not guaranteed or endorsed by the publisher.

## Supplementary Material

Supplementary Table S1 This table provides details on the type, frequency, and measurements collected for each survey.

## Abbreviations

AU: American University
BR: back reef
FR: fore reef
HIES: Household Income and Expenditure Survey
HSPH: Harvard T.H. Chan School of Public Health
KNSO: Kiribati National Statistics Office
LR: lagoon reef
MFMRD: Ministry of Fisheries and Marine Resource Development
MHMS: Ministry of Health and Medical Services
MT: manta tow
SIQ: soft-infaunal quadrat
SPC, Pacific Community: formerly known as the Secretariat of the Pacific Community
UCSB, University of California: Santa Barbara
UCSC, University of California: Santa Cruz
UW: University of Wollongong
USD: United States Dollar
GDP: Gross domestic product.
